# Patients with Incompetent Valves in Chronic Venous Insufficiency Show Increased Systematic Lipid Peroxidation and Cellular Oxidative Stress Markers

**DOI:** 10.1155/2019/5164576

**Published:** 2019-06-10

**Authors:** Miguel A. Ortega, Beatriz Romero, Ángel Asúnsolo, Mayte Sola, María J. Álavrez-Rocha, Felipe Sainz, Melchor Álavrez-Mon, Julia Buján, Natalio García-Honduvilla

**Affiliations:** ^1^Department of Medicine and Medical Specialties, Faculty of Medicine and Health Sciences, University of Alcalá, and Networking Biomedical Research Center on Bioengineering, Biomaterials and Nanomedicine (CIBER-BBN), Ramón y Cajal Institute of Sanitary Research (IRYCIS), Madrid, Spain; ^2^Department of Surgery, Medical and Social Sciences, Faculty of Medicine and Health Sciences, University of Alcala, and Ramón y Cajal Institute of Sanitary Research (IRYCIS), Madrid, Spain; ^3^Angiology and Vascular Surgery Service, Central University Hospital of Defense-UAH, Madrid, Spain; ^4^Immune System Diseases-Rheumatology and Oncology Service, University Hospital Príncipe de Asturias, Alcalá de Henares, Madrid, Spain

## Abstract

Chronic venous insufficiency (CVI) is a disease that impacts cellular homeostasis. CVI may occur with a valvular destruction process known as venous reflux or valvular incompetence. One of the cellular processes that may be triggered as a consequence of these events is the production of reactive oxygen species (ROS), which may trigger the production of different cellular markers and cell damage processes, such as lipid peroxidation. Therefore, the present study performed an observational, analytical, and prospective cohort study by reviewing 110 patients with CVI, and the activities and plasma levels of iNOS, eNOS, NOX1, and NOX2 were determined using immunohistochemistry and RT-qPCR. Lipid peroxidation (MDA) was also measured. Patients were distributed according to the presence or absence of valvular incompetence-venous reflux, which was diagnosed clinically as the absence of venous reflux (NR = 29) or presence of venous reflux (R = 81). Each group was divided according to age, with a cutoff point of fifty years (NR < 50 = 13, NR ≥ 50 = 16, R < 50 = 32, and R ≥ 50 = 49). The results showed that R patients exhibited significantly increased plasma MDA levels, and R < 50 patients exhibited the highest statistically significant increase. iNOS, NOX1, and NOX2 exhibited the highest gene and protein expression in R patients. The increased expression was maintained in the R < 50 patients. Our data suggest that young patients with valvular incompetence (venous reflux) show higher levels of lipid peroxidation and oxidative stress, which reflects the characteristics of an aged patient.

## 1. Introduction

Chronic venous insufficiency (CVI) is a disorder of the venous system that prevents the return of blood to the heart [1]. In general, IVC is not a serious pathology, but it occurs with a high incidence in the population [2, 3]. Currently available pharmacological treatments are not effective, and surgery is the treatment of choice when the disease progresses. In fact, these patients represent one of the most common consultations to vascular surgeons [4]. Different epidemiological studies performed worldwide reveal that CVI is a chronic pathology that occurs with high incidence and prevalence in the population [5, 6]. One of the main risk factors for developing CVI is age because of the progressive deterioration of the venous wall and increased pressure at the level of the superficial venous system. Other factors that influence the development of CVI are gender, family history, ethnicity, number of pregnancies, obesity, and risk professions [7–11]. CVI is associated with a wide variety of signs and symptoms, but it seems likely that all of the symptoms are related to venous hypertension. Venous hypertension often occurs due to reflux caused by incompetent venous valves [12]. These valves decrease the venous pressure, which favors the return of blood to the heart, and tolerate high pressures for limited periods of time. Therefore, events that modify the structure of these valves will trigger valvular incompetence and generate a blood reflux that progressively increases the venous pressure in the leg [13].

Reactive oxygen species (ROS) are physiologically produced in a regulated manner from the incomplete reduction of oxygen in the vascular wall. An imbalance between the production of ROS and the antioxidant defense mechanisms creates an oxidative stress that produces lipid peroxidation, oxidation of DNA, RNA, protein, and the inactivation of some enzymes [14–16]. Numerous authors demonstrated that the roles of nitric oxide (NO) and nitric oxide synthase (NOS) in vascular diseases are prominent in ROS activity [1, 17, 18].

The present study examined the process of valvular incompetence (venous reflux) and measured the differential expression of cellular oxidative stress markers (iNOS, eNOS, NOX1, and NOX2) according to patient age and how these conditions change the profile of lipid peroxidation as quantified using malondialdehyde (MDA). The aim of this study is to demonstrate how the oxidative stress that occurs at the tissue level has systemic consequences in correlation with age.

## 2. Patients and Methods

### 2.1. Study Population

This study was an observational, analytical, and prospective cohort study that reviewed patients with chronic venous insufficiency. Patients were divided according to age (cutoff point at 50 years of age) and the presence (R) or absence (NR) of incompetent valves (venous reflux). There were a total of 110 patients [NR = 29, 51.51 ± 14.04 years (NR < 50 = 13, 38.53 ± 6.21 years, NR ≥ 16, 62.06 ± 8.54 years), R = 81, 50.09 ± 15.91 years (R < 50 = 32, 62.06 ± 8.54 years, R ≥ 49, 59.98 ± 11.81 years)]. The study cohort was selected according to the following criteria. *Inclusion criteria:* women and men diagnosed with CVI and with and without venous reflux in the great saphenous vein; BMI ≤ 25; informed consent signed; and commitment to follow-ups during the pre- and postoperative periods plus tissue sample collection. *Exclusion criteria:* patients with venous malformations or arterial insufficiency; patients who did not provide their clinical history; patients with pathology affecting the cardiovascular system (e.g., infectious diseases, diabetes, dyslipidemia, hypertension); patients with toxic habits; and patients who doubted that they could complete the full follow-up.

Each patient underwent an exploratory examination using a M-Turbo Eco-Doppler (SonoSite) transducer of 7.5 Mz. The examination of the lower limbs was performed in a standing position with the explored leg in external rotation and support on the contralateral leg. The examination included the greater saphenous axis from the inguinal region to the ankle and femoral vein. A distal compression maneuver was performed. Valsalva maneuvers were performed in the present study. Pathological reflux was considered when this was greater than 0.5 sec. NR patients had a compressive syndrome as the indication for surgery. Patients were classified according to CEAP international criteria [18].

Saphenectomy of the vein was produced, and the total of the arch of the greater saphenous vein was taken. These fragments were introduced into two different sterile tubes: one tube contained minimum essential medium (MEM) with 1% antibiotic/antimycotic (both from Thermo Fisher Scientific, Waltham, MA, USA) and the other tube contained RNAlater® solution (Ambion, Austin, TX, USA). Blood samples are taken from the study population via puncture of the superficial vein of the elbow fold, after placement of a tourniquet on the arm. One tube (Vacutest® Kima, Piove di Sacco, Italy) of blood sample was collected from each study subject. The tube contained heparin to obtain blood serum.

The present study was performed in accordance with the basic ethical principles, autonomy, beneficence, nonmaleficence, and distributive justice, and its development followed Good Clinical Practice standards and the principles enunciated in the last Declaration of Helsinki (2013) and the Convention of Oviedo (1997). Patients were duly informed, and each was asked to provide written informed consent.

### 2.2. RT-qPCR

RNA was extracted from the samples collected in RNAlater® using the guanidine-phenol-chloroform isothiocyanate method of Chomczynski and Sacchi (1987). RNA samples (50 ng/*μ*l) were used to synthesize complementary DNA (cDNA) via reverse transcription. Each sample (4 *μ*l) was mixed with 4 *μ*l of an oligo-dT solution (15) 0.25 *μ*g/*μ*l (Thermo Fisher Scientific) and incubated at 65°C for 10 minutes in a dry bath (AccuBlock™, Labnet International, Inc., Edison, NJ, USA) to denature the RNA, following the protocol of Ortega et al. [3]. The amount of cDNA in each sample of the following genes of interest was quantified using qPCR. De novo primers or specific primers were designed for all of the genes studied (Table 1) using the Primer-BLAST online application [19] and AutoDimer [20]. The constitutively expressed genes of glyceraldehyde 3-phosphate dehydrogenase (GAPDH) were used to formalize the results. Gene expression was normalized using GAPDH as reference gene. The qPCR was performed in a StepOnePlus™ System (Thermo Fisher Scientific), and the relative standard curve method was used. For this, 5 *μ*l of each sample was mixed 1/20 with 10 *μ*l of iQ™ SYBR® Green Supermix (Bio-Rad Laboratories), 1 *μ*l of forward primer, 1 *μ*l of reverse primer (reverse primer), and 3 *μ*l of DNase and RNase-free water in a MicroAmp® 96-well plate (Thermo Fisher Scientific), for a total reaction volume of 20 *μ*l. Fluorescence detection was performed at the end of each repetition cycle (amplification) and at each step of the dissociation curve. The data obtained from each gene were interpolated using a standard curve created from serial dilutions of a mixture of the study samples that was included in each plate. Results are expressed as arbitrary units. All tests were performed in duplicate.

### 2.3. Immunohistochemistry

Samples destined for immunohistochemical studies were processed using standardized protocols [3, 21]. Samples were embedded in paraffin and sectioned using a microtome into 5 *μ*m thick sections. Sections were deparaffinized and hydrated. The different study molecules were detected using commercial primary and secondary antibodies (Table 2). Sections of the same tissue were used as negative controls in all immunohistochemical studies, in which the primary antibody was replaced with blocking solution. Detection of the antigen-antibody reaction was performed using the ABC method (avidin-biotin complex) (DAB Kit, SK-4100, Vector, Burlingame, CA, USA), which used the chromogen avidin-peroxidase ExtrAvidin®-Peroxidase (Sigma-Aldrich, St. Louis, MO, USA) at a 1 : 200 dilution in PBS.

Histological samples of the patients were stratified as negative (0) or positive (1). For each of the patients of the established groups, 5 sections and 5 random fields per section were examined. Patients were described as positive when the average of the test sample marked for each study subject was greater than or equal to 5% of the total [22].

### 2.4. Oxidative Stress Determination

MDA production is proportional to polyunsaturated fatty acid degradation of lipid peroxidation. Therefore, MDA concentration was measured to determine the oxidative stress in patient plasma. The lipid peroxidation assay kit (ab118970) is a suitable method for the sensitive detection of the malondialdehyde of the sample. The MDA present in the sample reacts with thiobarbituric acid (TBA) to generate an MDA-TBA adduct, which is easily quantified using colorimetry. The sensitivity of this method was 0.1 nmol MDA/well.

### 2.5. Statistical Analysis

GraphPad Prism 5.1 software was used for statistical analyses, and the Mann-Whitney *U* test was used. Data are expressed as the means ± standard deviation. The significance was set at *p* < 0.05 (∗), *p* < 0.005 (∗∗), and *p* < 0.001 (∗∗∗).

## 3. Results

### 3.1. Study of Lipid Peroxidation Levels: Malondialdehyde

**Figure 1:**
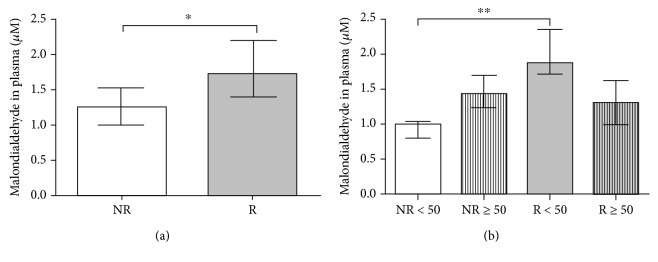
(a) Diagram showing the levels of malondialdehyde (MDA) in *μ*M in the plasma of patients without reflux (NR) and with reflux (R). ∗*p* < 0.05. (b) Diagram showing the *μ*M levels for malondialdehyde (MDA) in the plasma of patients without reflux less than fifty years of age (NR < 50), without reflux greater than or equal to fifty years of age (NR ≥ 50), with reflux less than fifty years of age (R < 50), and with reflux greater than or equal to fifty years of age (NR ≥ 50). ∗∗*p* < 0.005.

Lipid peroxidation levels were determined using malondialdehyde levels in the plasma of the study cohort. Patients with venous reflux (R) exhibited a significant increase compared 1to the NR subjects (*p* < 0.05) (Figure 1(a)). The mean malondialdehyde levels were 1.306 ± 0.116 *μ*M in nonreflux patients and 1.745 ± 0.142 *μ*M in patients with reflux. A clear differential distribution was found in relation to the age factor, which significantly increased the levels of malondialdehyde in R < 50 patients compared to NR < 50 patients (0.952 ± 0.067 *μ*M, NR < 50 versus 1.966 ± 0.142 *μ*M, R < 50), *p* < 0.005 (Figure 1(b)). No significant differences were observed between groups greater than or equal to fifty years of age (1.508 ± 0.124 *μ*M, NR ≥ 50 versus 1.303 ± 0.175 *μ*M, R ≥ 50).

### 3.2. iNOS and eNOS

**Figure 2:**
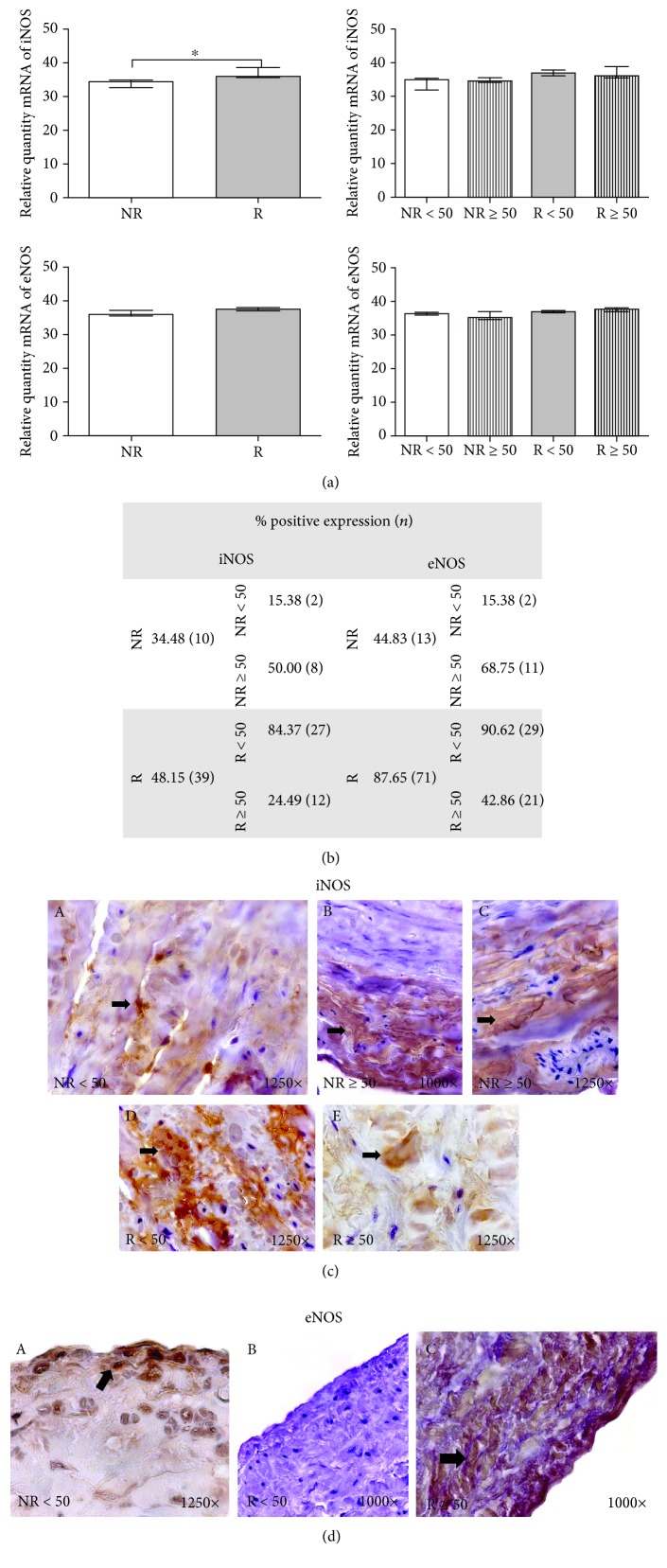
(a) Levels of mRNA for iNOS and eNOS quantified using RT-qPCR in patients without reflux (NR) and with reflux (R) and by their ages. Results are expressed as arbitrary units. ∗*p* < 0.05. (b) Distribution of the percentage of patients with positive protein expression for iNOS and eNOS in patients without reflux (NR) and with reflux (R) and by age, *n* = number of patients. (c) Protein expression images of iNOS in NR < 50 (A), NR ≥ 50 (B, C), R < 50 (D), and R ≥ 50 (E) patients. (d) Protein expression images of eNOS in NR < 50 (A), R < 50 (B), and R ≥ 50 (C) patients. The arrows are the brown coloration indicating the specific precipitate that correlates with the expression of the said protein.

The gene expression measure of iNOS was 34.168 ± 1.424 in NR versus 36.665 ± 2.314 in R, which was significantly different (*p* < 0.05). The distributions of gene expression by age were 34.209 ± 2.113 in NR < 50 versus 34.127 ± 0.773 in NR ≥ 50 and 36.536 ± 1.977 in R < 50 versus 36.730 ± 2.758 in R ≥ 50. No significant differences in eNOS were obtained (36.090 ± 1.164 in NR versus 37.703 ± 0.889 in R) (Figure 2(a)).

The study patients exhibited differential protein expression of iNOS and eNOS (Figure 2(b)). These markers represented 34.48% and 44.83% in NR patients, respectively. These values were 48.15% and 61.73%, respectively, in R patients. There was a marked increase in the number of R patients who exhibited positive protein expression.

When the age factor was considered, the values of iNOS were 15.38% in NR < 50 and 50.00% in NR ≥ 50 patients. These values were 84.37% for R < 50 and 24.49% for R ≥ 50 patients. The expression of eNOS was 15.38% in NR < 50 and 68.75% in R ≥ 50. At reflux, eNOS was 90.62% in R < 50 compared to 42.86% in R ≥ 50. These results show that NR ≥ 50 and R < 50 patients exhibited the highest percentage of positive expression for iNOS and eNOS.

iNOS expression showed that marker differences were established in the different layers of the human vein according to patient age (Figure 2(c)). iNOS protein was clustered in the three tunicas of NR patients. However, NR ≥ 50 patients exhibited a greater intensity of protein expression that was located more intensely in the adventitial tunica (Figure 2(c), B and C). NR < 50 patients exhibited large accumulations along the entire length of the vein wall, which was very intense in the middle tunica (Figure 2(c), D and C). The expression of eNOS was differentially maintained in the endothelium of NR < 50 patients, and it was especially intense in the adventitial tunica of R ≥ 50 patients (Figure 2(d), A–C).

### 3.3. NOX1 and NOX2

**Figure 3:**
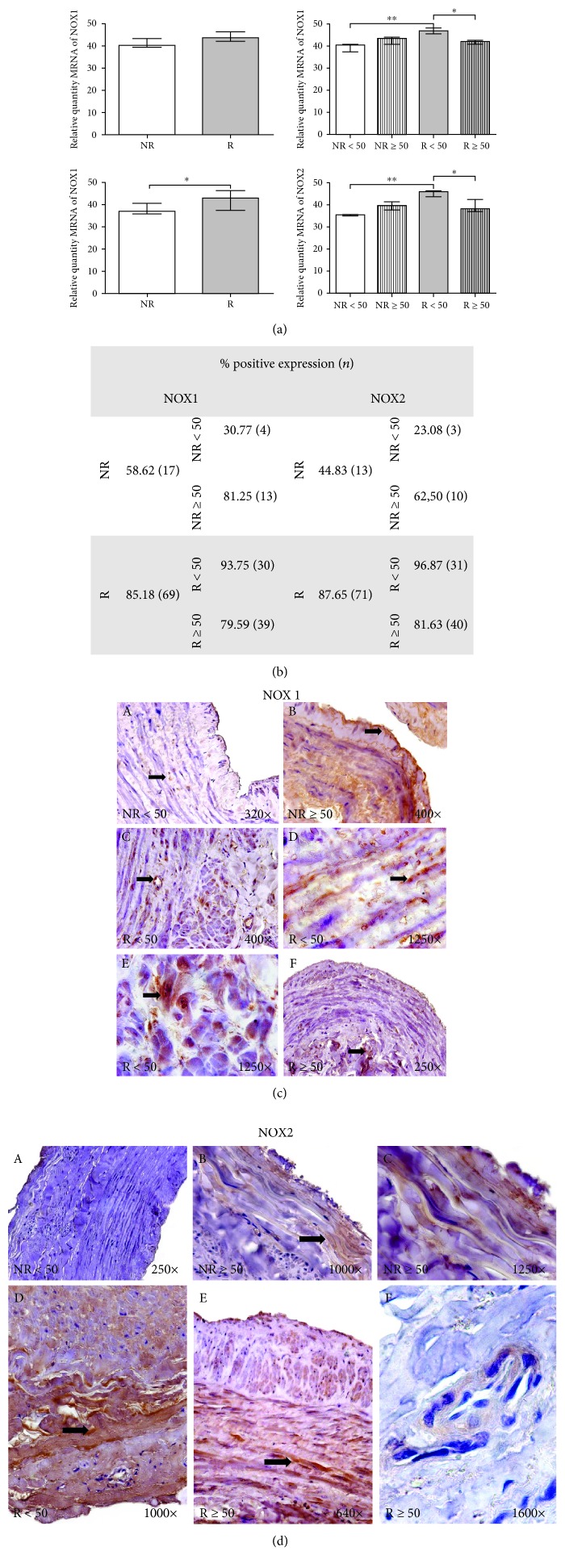
(a) RNA levels of NOX1 and NOX2 quantified using RT-qPCR in patients without reflux (NR) and with reflux (R) and by the ages of the same groups. Results are expressed as arbitrary units. ∗*p* < 0.05, ∗∗*p* < 0.005. (b) Distribution of the percentage of patients with positive protein expression for NOX1 and NOX2 in patients without reflux (NR) and with reflux (R), and by age, *n* = number of patients. (c) Protein expression images of NOX1 in NR < 50 (A), NR ≥ 50 (B), R < 50 (C, D, E), and R ≥ 50 (F) patients. (d) Protein expression images of NOX2 in NR < 50 (A), NR ≥ 50 (B, C), R < 50 (D), and R ≥ 50 (E, F) patients. The arrows are the brown coloration indicating the specific precipitate that correlates with the expression of the said protein.

NOX1 gene expression analysis did not reveal any significant differences between the study groups (40.704 ± 2.534 in NR versus 43.943 ± 2.842 in R). The distributions of gene expression by age were 38.942 ± 1.792 in NR < 50 versus 42.465 ± 1.879 in NR ≥ 50 and 46.408 ± 1.140 in R < 50 versus 41.479 ± 0.821 in R ≥ 50 (*p* < 0.05). Statistically significant differences were established between NR < 50 and R < 50 patients (*p* < 0.005) (Figure 3(a)).

An increase in NOX2 gene expression was observed in patients with venous reflux (37.686 ± 2.643 in NR versus 42.015 ± 4.011 in R) (*p* < 0.05). The distributions of gene expression by age were 35.022 ± 0.296 in NR < 50 versus 39.018 ± 2.125 in NR ≥ 50 and 45.136 ± 1.582 in R < 50 versus 38.894 ± 2.015 in R ≥ 50 (∗*p* < 0.05) (Figure 3(a)).

NOX1 and NOX2 proteins were differentially expressed in the established groups. For NOX1 expression, 58.62% of NR patients were positive compared to 85.18% of R patients. NOX2 expression was positive with 44.83% and 87.65%, respectively (Figure 3(b)). When studying the distribution of this expression as a function of age, it was observed that R < 50 patients exhibited the highest percentages of NOX1 and NOX2 (93.75% and 96.87%, respectively).

The study of the distribution of expression in the different layers of the human vein revealed important data on histological compression. NR ≥ 50 and R < 50 patients exhibited higher NOX1 protein expression in the intima, media, and adventitia layers of the human vein, and these differences were statistically significant (Figure 3(c), A–F).

NOX2 protein expression was increased in R patients compared to NR patients in the intima, media, and adventitia layers of the vein. R < 50 patients showed a greater intensity of expression in the three tunicas of the venous wall (Figure 3(d), A–F).

## 4. Discussion

The multitude of mechanisms involved in the progression of CVI made it difficult for the scientific community to identify the factors that trigger this disease. Some studies related reflux with weakening of the venous walls [23], which may be due to an imbalance in the content of collagen and elastin in the vein [24]. Other studies focused on chronic inflammation as the main factor for the onset of the pathology [25]. Krzysciak and Kózka [26] showed that oxidative stress increased the risk of damage to the vascular endothelial wall and DNA and caused a remodeling of the tissue and the consequent progression of the pathology. Therefore, one of the events involved in valvular incompetence is oxidative stress.

Krzysciak and Kózka [26] mentioned that ROS promotes reflux that generates a hypoxic environment in endothelial cells. These events favor the adhesion of leukocytes and other inflammatory mediators that release angiotensin II, which exerts a vasoconstrictive action directly on the smooth muscle and is capable of increasing the expression of growth factors, matrix metalloproteinases (MMPs), and collagen [1, 27]. Overexpression of MMPs was also observed in fibroblasts, endothelial cells, and smooth muscle cells in patients with CVI [28]. Therefore, an alteration in cell balance may cause degenerative damage that compromises cell structure, the content of collagen and elastin, and the contraction and relaxation properties of the smooth muscle of the venous wall [29].

Therefore, ROS plays a decisive role in the progression of chronic venous insufficiency. Our results showed that R < 50 patients exhibited the highest concentrations of MDA in plasma. Krzysciak and Kózka [26] measured MDA concentrations in samples of saphenous veins of patients with CVI before and after development of the disease. These results showed a relationship between oxidative stress and chronic venous insufficiency at the tissue level and the systemic level beginning in the first years of the disease. Mikuła-Pietrasik et al. [30] showed that the sera of varicose patients increased cell proliferation, expression of the senescence marker SA-*β*-Gal, and ROS production in the endothelial cells of human umbilical veins (HUVECs) compared to the sera of healthy individuals. This result suggests that the presence of oxidative stress at a systemic level is the main factor triggering the progression of the pathology.

Angiotensin II also activates nicotinamide adenine dinucleotide phosphate (NADPH) oxidase and enhances the production of superoxide anion (O_2_^−^) due to endothelium wall stress-dependent stimulation [26]. In addition to being a vasoconstrictor substance, it promotes inflammation, hypertrophy, and fibrosis, and it is implicated in vascular damage and remodeling in cardiovascular diseases [31]. A recent study by Zhang et al. [31] showed that an increase in the expression of NOX1 and NOX2 occurred after the stimulation with angiotensin II in HUVECs. Our results showed the event of oxidative stress in relation to NOX1 and NOX2 and the existence of a differential expression based on the age of the patients. These results should make us consider the implication of an accelerated aging process that leads to greater oxidative and inflammatory stress in the valvular incompetence (venous reflux). In fact, numerous authors noted the correlation of oxidative stress with age, but an accelerated aging process was not mentioned in young patients [22, 32]. On the other hand, we wanted to further develop the implications of iNOS and eNOS in chronic venous insufficiency because many authors mentioned the role that these molecules play in vascular diseases [33].

eNOS is expressed primarily in endothelial cells. Therefore, our immunohistochemistry images of the low expression of eNOS in the tunica intima of the veins of patients with reflux stand out compared to patients without reflux. By providing a baseline level of NO in the vein and neutralizing ROS, it makes sense that patients with low eNOS expression are more susceptible to endothelial deterioration and develop valvular incompetence (venous reflux). The low expression of eNOS may be related to CVI and any disease in which the mechanism involves endothelium dysfunction, as indicated by Mikuła-Pietrasik et al. [30]. However, the expression of eNOS in the tunica adventitia suggests that it is reactive and remains functionally active. Our studies found differences in the iNOS isoform in the adventitia and middle vein tunicas. NR ≥ 50 patients tended to exhibit an increase in iNOS expression in the adventitia tunica, likely in response to age-induced stress. Notably, the expression of iNOS in patients with reflux never reached the expression detected in NR ≥ 50 patients, despite the oxidative stress generated in these patients. The low expression of eNOS and iNOS decreases the bioavailability of NO in the vein, which makes it more susceptible to oxidative stress. However, the increase in iNOS expression is related to other cardiovascular pathologies [34]. The decrease in the expression of iNOS and eNOS suggests the existence of a suppressive mechanism of expression, perhaps at the level of protein transcription because both proteins are encoded by different genes but share a 50-60% homology in amino acid sequence [35]. Our results support a role for oxidative stress as a mechanism involved in the development of valvular incompetence (venous reflux) in CVI. The present study showed the existence of an oxidative environment in human veins with chronic venous insufficiency and how the different molecular components that participate in CVI were differentially expressed in correlation with the age of the patients. Our study presents some limitations, since to observe the tissue response it would be necessary to develop *in vitro* experiments of the endothelial and muscle cells of the saphenous vein. In this line, another limitation of our study is to observe if this profile of protein and gene expression is the same in other venous territories of the lower limb. However, our study is the first to show how valvular incompetence has important consequences and there is a different profile depending on age.

The importance of this study lies in demonstrating how venous disease produces a tissue change with systemic consequences. Venous disease is a common pathology in the general population that produces great disabilities, knowing its pathophysiology and its systemic consequences will help the development of specific therapies. Future studies should be aimed at discovering possible therapeutic targets at the tissue level that prevent systemic change and its consequences.

## Figures and Tables

**Table 1:** The primers used in RT-qPCR, the sequence, and the binding temperature (Temp).

Gene	Sequence fwd (5′→3′)	Sequence rev (5′→3′)	Temp
GADPH	GGA AGG TGA AGG TCG GAG TCA	GTC ATT GAT GGC AAC AAT ATC CAC T	60°C
eNOS	AAG AGG AAG GAG TCC AGT AAC ACA GA	ACG AGC AAA GGC GCA GAA	60°C
iNOS	CCT TAC GAG GCG AAG AAG GAC AG	CAG TTT GAG AGA GGA GGC TCC G	61°C
NOX1	GTT TTA CCG CTC CCA GCA GAA	GGA TGC CAT TCC AGG AGA GAG	55°C
NOX2	TCC GCA TCG TTG GGG ACT GGA	CCA AAG GGC CCA TCA ACC GCT	60°C

**Table 2:** Primary and secondary antibodies used in the immunohistochemical studies performed, showing the dilutions used and the specificities in their protocol.

Antigen	Species	Dilution	Provider	Protocol specifications
eNOS	Rabbit	1:100	Abcam (ab66127)	Citrate tampon in heat (pH = 6)
iNOS	Rabbit	1:500	Abcam (ab95866)	—
NOX1	Rabbit	1:250	Abcam (ab78016)	EDTA (pH = 9) before incubation with blocking solution
NOX2	Goat	1:500	Abcam (ab111175)	—
Anti-rabbit IgG	Mouse	1:1000	RG-96 (Sigma-Aldrich)	—
Anti-goat IgG	Mouse	1:100	A5420 (Sigma-Aldrich)	—

## Data Availability

The data used to support the findings of the present study are available from the corresponding author upon request.
